# IKVAV-linked cell membrane-spanning peptide treatment induces neuronal reactivation following spinal cord injury

**DOI:** 10.4155/fso.15.81

**Published:** 2015-11-01

**Authors:** Soheila Kazemi, Wendy Baltzer, Karl Schilke, Hadi Mansouri, John Enrique Mata

**Affiliations:** 1 Department of Clinical Sciences, College of Veterinary Medicine, Oregon State University, Corvallis, OR, USA; 2 School of Chemical, Biological & Environmental Engineering, Oregon State University, Corvallis, OR, USA; 3 Department of Biomedical Sciences, College of Veterinary Medicine, Oregon State University, Corvallis, OR, USA; 4 Department of Basic Medical Science, Western University of Health Sciences, Pomona, CA, USA

**Keywords:** spinal cord injury, regeneration, IKVAV, regeneration, membrane-spanning peptide

## Abstract

Spinal cord regeneration following treatment with a novel membrane-spanning peptide (MSP) expressing the isoleucine-lysine-valine-alanine-valine (IKVAV) epitope was assessed in Balb-c mice. After hemilaminectomy and compression injury, mice were treated with IKVAV, IKVAV-MSP, peptide or vehicle control. Functional improvement was assessed using modified Basso, Beattie, and Bresnahan Scale (mBBB) and spinal cord segments were studied histologically 28 days after injury. IKVAV-MSP group scores increased significantly compared with control groups after 4 weeks of observation (p < 0.05). The number of protoplasmic astrocytes, neurons and muscle bundle size in the IKVAV-MSP mice were significantly increased (p < 0.001; p < 0.05 and p < 0.007; respectively). This study demonstrates that it is possible to promote functional recovery after SCI using bioactive IKVAV presenting cell membrane-spanning peptides.

## Abstract (lay abstract)

**Lay abstract:** A new potential treatment for spinal cord injury was tested in mice. Mice were treated with our membrane-spanning peptide with the IKVAV motif (IKVAV-MSP) or various control treatments. Functional improvement was assessed daily and spinal cord segments were evaluated after 4 weeks. IKVAV-MSP treatment significantly restored function compared with control groups, numbers of health nerve cells within the spinal cord were increased and muscles appeared healthier. This study demonstrates that it is possible to promote functional recovery after SCI using bioactive IKVAV presenting cell membrane-spanning peptides.

**Figure 1.**
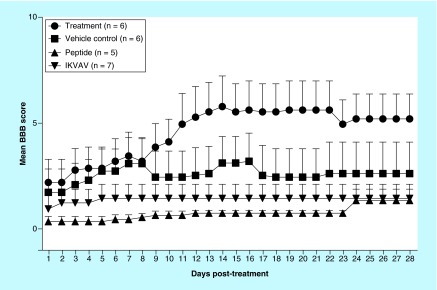
**Effects of IKVAV-membrane-spanning peptide treatment on neurological status with daily assessment for 28 days following spinal cord clip compression injury.** Two-way ANOVA followed by Tukey’s multiple comparison test revealed a significant improvement in locomotor control compared with peptide alone on days 11–28 (p ≤ 0.05) and IKVAV alone on days 11–28 (p ≤ 0.05).

**Figure 2.**
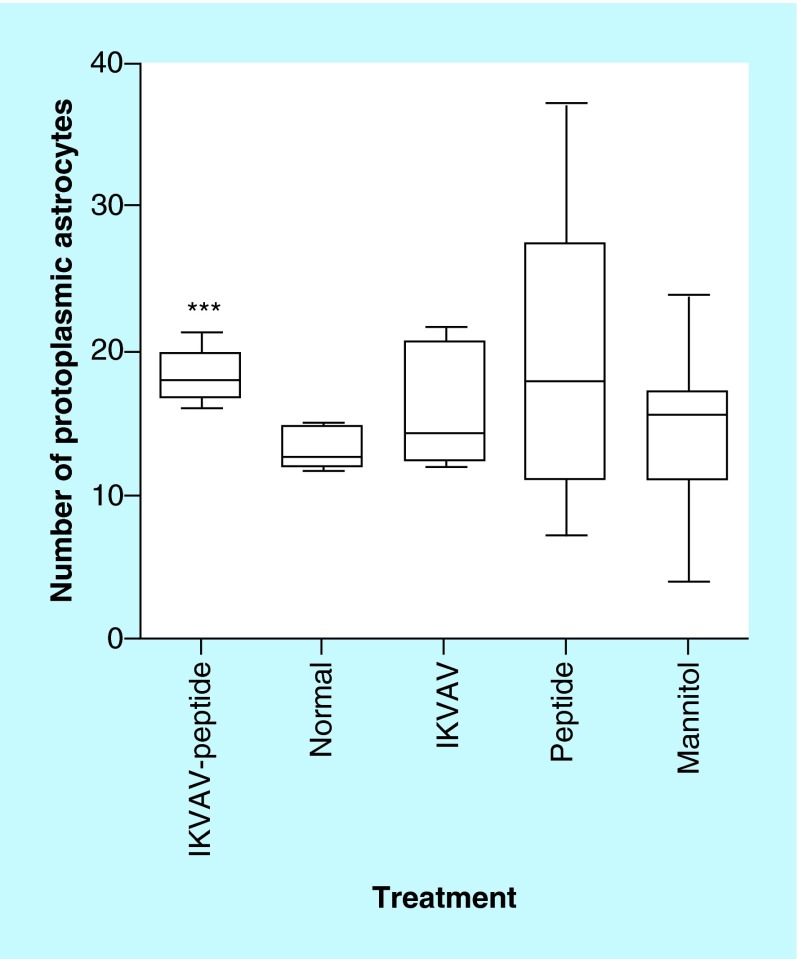
**Effects of IKVAV-membrane-spanning peptide treatment on protoplasmic astrocyte number within the area of injury for each group of mice.** Three fields were viewed, enumerated and averaged for each mouse. *** denotes significantly increased numbers of astrocytes compared with all groups except the Peptide group (p < 0.001).

**Figure 3.**
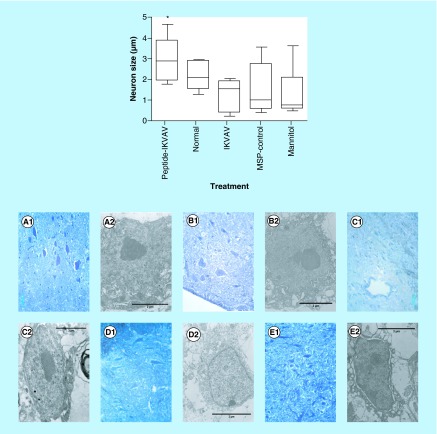
**Effects of IKVAV-membrane-spanning peptide treatment on neuron size.** Graph shows mean neuron size at 4 weeks after SCI for all animals determined by measurement of 6 neurons per mouse within the region of interest. * denotes that IKVAV-MSP differed from all others at p < 0.05. The pictures presented in A-E shows the comparison between representative groups at light under 400X magnification (1) and electron microscope (2) level. In IKVAV-Peptide group **(A)**, the large neurons are obvious and actually larger compared with Normal **(B);** however, in control groups IKVAV **(C)**, Peptide **(D)** and Mannitol **(E)** the neurons are shrunken and in some cases pyknotic.

**Figure 4.**
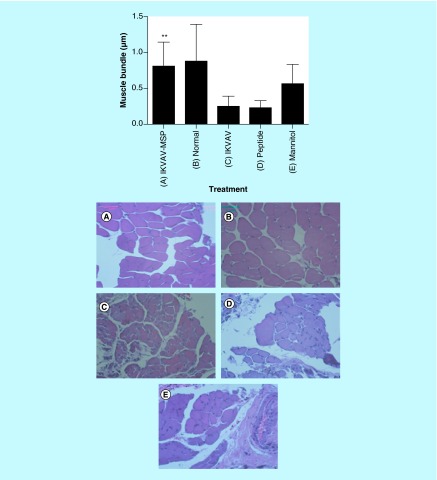
**Effects of IKVAV-MSP on muscle bundle size determined from enumeration of 10 fields from both right and left legs for each mouse.** ** denotes a significant difference between representative treatment and control groups (p < 0.01). Comparison of muscle bundles in IKVAV-MSP treated **(A)**, normal untreated **(B)** and control treated groups (**C–E**, IKVAV, Peptide and Mannitol treatment, respectively). Histological images are H&E X400.

Spinal cord injury (SCI) is a debilitating condition that stimulates complex cellular and molecular interactions in the spinal cord. These interactions include contributions from inflammatory cell activation, reactive astrocytes and the production of both growth promoting and inhibitory extracellular molecules [1]. The cascade of events that occur after SCI at the site of injury often results in incomplete repair of damaged tissue with altered cellular morphology and loss of function. Biological analysis of SCI in mouse models has pointed to both necrotic and apoptotic mechanisms of cell death after injury [2]. Electron micrographs of neurons in gray matter following SCI have shown fragmented nuclei and condensed bodies along with shrunken cell bodies. Neuronal and glial apoptosis following SCI can contribute to neurological dysfunction, paresis and paralysis [3]. Spinal cord injury also results in glial reaction which recruits microglia, oligodendrocyte precursors, meningeal cells, astrocytes and stem cells.

Activated astrocytes migrate and eventually form a glial scar at the site of the injury which inhibits axon regeneration and recovery of normal function. The glial scar that forms following spinal injury is an evolving structure, with different cells arriving and participating at different stages, to create a final structure that is predominantly astrocytic. This astrocyte response to injury is referred to as reactive gliosis (more glia) but in fact, in most instances of SCI, the actual amount of glial cell division is relatively small and confined to the immediate penumbra surrounding the lesion core [4–8]. Astrocytes form the majority of the scar and may be responsible for inhibition of neuron regeneration across the site of injury.

We hypothesize that intervention leading to successful regeneration of neuronal tissue within the injured spinal cord will reduce the astrocyte response that contributes to scar formation. Further, more complete regeneration guided by sustained cellular signals will likely result in morphologically normal cellular architecture that will be evident by TEM. To test this hypothesis, we chose a transmembrane-spanning peptide (MSP) that acts as a molecular anchor and presents the IKVAV motif when applied to injured tissue. IKVAV is an active sequence of laminin–1 which is located in the C-terminal end of the long arm of the α1 chain which promotes cell adhesion, neurite outgrowth, angiogenesis, collagenase IV production and tumor growth [9–13]. In a recent study [14], treatment with IKVAV PA (peptide amphiphile) following SCI resulted in reduced astrogliosis and subsequent glial scar as well as reduced cell death. IKVAV also increased the number of oligodendroglia, promoted regeneration of descending motor fibers and ascending sensory fibers and resulted in significant behavioral improvement experimentally in mice.

The specific purposes of this study were to evaluate the response to the IKVAV-MSP within the site of compression injury to elucidate the changes in cellular architecture compared with normal and untreated tissue. We analyzed nerve cells, astrocytes and glial scar formation in the injured spinal cord. In addition, we determined the effect of IKVA-peptide on the rate of muscle atrophy following SCI.

## Materials & methods

### Animal care & mouse spinal cord injury

All animal procedures were undertaken in accordance with the Public Health Service Policy on Human Care and Use of Laboratory Animals. All procedures were approved by the Institutional Animal Care and Use Committee (IACUC) at Oregon State University. Forty-three healthy skeletally mature Female Balb-c mice (10 weeks of age) were divided into five groups of 7–9 animals each with a nonsurgical group of six normal (untreated group) mice and surgical treatment groups including: vehicle (Mannitol 0.5 mg/ml), control (IKVAV 0.5 mg/ml), control (Peptide, AELLLELELLELELLL, 0.5 mg/ml) and treatment (IKVAV-MSP, AELLLELELLELELLLAIKVAV, 0.5 mg/ml). All peptides were synthesized with 95% purity. We anticipated needing six mice per treatment group based on analysis of preliminary data; however, this number was increased to account for animal loss unrelated to treatment that included three animals in the treatment group removed for excessive chewing of limbs as they regained function, three in the control group and three in the vehicle group that died during the first week, and two in the vehicle group that showed no sign of injury. Animals were anesthetized with isoflurane inhalant (4–5% induction, 1–2% maintenance). After laminectomy, at the level of T12 vertebral segment, the spinal cord was compressed dorsoventrally by the extradural application of a 24 g modified aneurysm clip for 1 min [15]. Following spinal cord injury, the incision was closed using metal staples (Autosuture, Norwalk, CT). Postoperatively, animals were kept in cages placed on a circulating water pad (80°F) to prevent hypothermia and 1.0 ml injection of saline was administered once daily subcutaneously (s.c.) to prevent dehydration. Blocked bladders were manually emptied (by finger pressing the bladder over the pelvic region) two to three times daily throughout the study. We used phenoxybenzamine (5–10 mg/kg, orally) to address urine retention and bladder distention. Phenoxybenzamine is used to reduce internal urethral sphincter tone. It may also be used for urethral spasm secondary to bacterial urethritis [16]. Buprenorphine was given (0.05 mg/kg, s.c., twice daily) for analgesia in the event that the animal showed signs of discomfort (such as chewing, anorexia and other signs of pain). Enrofloxacin was administered once daily in the event of hematuria or self-mutilation (5 mg/kg) orally for 5–7 days. Two mice that exhibited any hind limb movement less than 24 h following the injury were excluded from the study.

### Peptide injection

IKVAV-MSP (0.5 mg/ml, 1% Phosphate Buffered Saline, 2.5% mannitol solution) or other treatments including control peptides at equal molar concentrations, were administered 24 h after injury using tuberculin syringes with a fine (25 gauge) needle. Under isoflurane anesthesia, the skin sutures were removed and the site of injury (subcutaneous portion) was exposed. A micropipette was inserted just under the granuloma formed at the site of injury dorsally on the spinal cord, and 10 µl of the IKVAV-MSP solution (approximately 5 µg total) or other treatments were applied to the injury site. The needle was carefully withdrawn and the wound was closed. For all experiments, the investigators were kept blind to the identity of the treatment groups.

#### Modified Basso, Beattie & Bresnahan Scale open-field locomotion score

To assess behavioral recovery from SCI, the modified Basso, Beattie and Bresnahan Scale (mBBB) open-field locomotion score was observed using the mBBB Locomotor Rating Scale which we found to be more discriminating for than the method described by Basso *et al.* [17–19]. For examination, the mice were placed individually in an open field on a non-slippery surface. The 22-point (0–21) mBBB scale was used to assess hind limb locomotor recovery including joint movements, stepping ability, coordination and trunk stability. A score of 21 indicates unimpaired locomotion as observed in uninjured mice whereas a score of 0 indicates complete absence of voluntary movement. The tests were performed by two examiners who were blinded to the animal’s treatments. Mice were assessed prior to injury and mBBB tests were performed every day after injury till the end of the study (28 days).

### Animal tissue acquisition & preparation

On day 28, animals were euthanized with an overdose intraperitoneal pentobarbital (beuthanasia-D, Schering–Plough Animal Health Corp., NJ, USA) 100 mg/kg (0.2 ml/mouse) and the spinal cords were removed after complete laminectomy at the site of the injury and immediately placed in 2% buffered formalin. The spinal cords were kept in the refrigerator overnight and then transferred to the electron microscopic laboratory for routine tissue processing. Semi-thin sections (0.5 µm) were cut using a Sorvall MT2–b (Norwalk, CT, USA) microtome for light microscopic observations and then ultrathin sections (60 nm) were cut with a diamond knife and put on 100 mesh copper grids for transmission electron microscopic observation using Philips CM/12 STEM (Eindhoven, Netherlands).

### Muscle preparation

Hamstring and quadriceps muscles were dissected from all groups and fixed in 2% buffered formalin for 24 h. Segments were embedded in paraffin and processed routinely for light microscopic evaluation of 5 µm sections stained with hematoxylin and eosin (H&E).

### Neuron & muscle bundle size quantification

The computer software ImageJ (Wayne Rasband, NIH, USA) was used to measure the neuron and muscle size. We used micrometer (µm) as a unit for measuring the surface of each neuron and each muscle bundle.

### Protoplasmic astrocyte quantification

Astrocytes were counted in three fields of each semithin section for each mouse at 400 magnification using calibrated ocular lens with Lattice lines.

#### Statistical analysis

Two-way ANOVA and general linear model were used to analyze the data. Differences in mBBB scores, body weight between group means and protoplasmic astrocytes numbers were analyzed using two-way ANOVA (mBBB analysis included a repeated measures test). Neuron and muscle size means were identified using linear model test and a significance level of p < 0.05. Statistical analysis was carried out using the open source statistical programming arrangement 'R' [20]. Statistical significance is noted with asterisk as * = p < 0.05; ** = p < 0.01; *** = p < 0.001.

## Results & discussion

To assess the functional recovery of the spinal cord and measure the basis of the functional recovery following injury, several outcome parameters were used including mBBB score, histological and electron microscopy. At 4 weeks after SCI, IKVAV-MSP mice displayed a significant improvement in neurological status (p < 0.05), whereas none of the other treatment groups were significantly altered from their immediate postoperative status (Figure 1).

### Effect of the IKVAV-MSP on protoplasmic astrocytes

As seen in Figure 1, IKVAV-MSP significantly improved neurological recovery following acute SCI, with improvements in motor function evident as early as 4 weeks following treatment. At 4 weeks following SCI, ocular counting of light-microscopic sections of injured spinal cords identified increased numbers of protoplasmic astrocytes in the IKVAV-MSP treatment group compared with IKVAV, Normal and Mannitol groups (p < 0.001) (Figures 1 & 2). However, IKVAV-MSP was not significantly different from the peptide-treated group (p > 0.05).

### Effect of the IKVAV-MSP on neuron size & neuron cell death

Injury-induced apoptosis can be detected from hours up to days following injury and may contribute to neurological dysfunction [21]. We used ImageJ software to measure the size of the neurons in each of the treatment groups to determine whether the injection of the IKVAV-MSP could support cellular regeneration of the motor neurons. The images of all sections were taken using a light microscope equipped with a digital camera (DFC280 Model, Leica Microsystems Ltd) [22], with 400 magnification. There was a significant increase in the size of the neurons of the gray matter in the IKVAV-MSP mice compared with the other four groups (p < 0.05, Figures 1, 2 & 3). Neuron reactivation as evinced by increase in metabolic activity was seen in the gray matter of the spinal cord at the site of injury. Neuron degeneration and death were detected using light microscope (LM) and transmission electron microscope (TEM) micrographs. The characteristics of neuronal death that were examined, included apoptosis and necrosis. These included shrunken cell bodies with intact cell membranes, chromatolysis evidence such as dispersed Nissl’s substances, and finally a loss of these cellular organelles throughout the cytoplasm along with loosely dispersed nuclear chromatin. Few expanded empty spaces were also noticed between the neuron cell bodies in IKVAV, Peptide and Mannitol mice. Using LM and TEM images we observed 1.4–2.4-folded larger neurons in the IKVAV-MSP group.

### Effect of the IKVAV-MSP on muscle size

Each section was photographed using digital camera connected to the light microscope, with 400 magnification. To measure the muscle bundles we used ImageJ software to assess the degree to which SCI with and without treatment can affect muscle morphology. The results of this measurement indicated that the size of muscle bundles in IKVAV-MSP-treated group was significantly increased compare with IKVAV and Peptide groups (p < 0.05) but not with Normal and Mannitol-treated mice (p > 0.05) (Figure 4). Control treated groups showed identified atrophied muscle bundles with considerable amount of muscular fat (Figure 4C–E).

### Effect of the IKVAV-MSP on body weight

The body weight was measured for each mouse daily, using a digital scale. Losses in body weight from prestudy values were similar between IKVAV-MSP (average loss of 4.4 ± 1.7 gm) and the other three groups with Vehicle, IKVAV and peptide groups lost an average of 4.9 ± 1.3, 6.0 ± 1.6 and 5.3 ± 2.4, respectively.

## Discussion

The pathophysiology of acute SCI involves primary and secondary mechanisms of injury [23–26]. These mechanisms involve the initial mechanical damage leading to secondary injury processes that contribute to further tissue loss, functional impairment, and lesion size increase [27,28]. Secondary injury involves the apoptotic and necrotic death of neurons and glial cells [29,30]. The microscopic evaluation of neuron death within the lesion site in this study is consistent with other studies, which have described similar amounts of apoptosis and necrotic death after SCI [3,21,30–33]. Although SCI leads to neuronal death, we have seen small numbers of surviving neurons with normal intact structure within the lesion site, which is consistent with other studies using the clamp model [34]. In the present study, IKVAV-MSP-treated mice revealed the largest number and size of neurons compared with all other groups (p < 0.05), thus indicating that the IKVAV-MSP induced a progressive metabolically active phase in these neurons to regain their normal function. However, in the other groups, reduction of neuron size characterized with shrunken cell cytoplasm may have represented necrotic and apoptotic phases of SCI, which is in agreement with other reports at 28 days postinjury [3].

The SCI was induced with the clamp crush method which is a technique relevant and similar to clinical spinal cord injury in humans [18]. In order to mimic clinical delays in definitive treatment while the patient is stabilized following SCI, we also chose to administer treatment approximately 24 h following surgery. Recovery was incomplete 28 days following treatment, however, IKVAV-MSP treatment promoted significant functional improvement that can be attributed to the observed differences in neuronal cell death and astrocyte function. Although reactive astrocytes may form a glial scar that can, in some cases, prevent axonal regeneration, they also have a crucial role in wound healing and functional recovery following SCI. Emergence and migration of reactive astrocytes have a prominent role in the repair of injured tissue and the restoration of motor function in the subacute phase of healing neuronal tissue before completion of the glial scar [35]. Loss of astrocyte activities during the cellular response to SCI can lead to gross functional deficits and failure of functional recovery and suggest that reactive astrocytes not only protect tissue but also preserve function after SCI [36,37].

In this experiment, IKVAV-MSP treatment improved neurological function and resulted in an increase in the number of astrocytes. Our results support the positive role of astrocytes in promoting neuron regeneration across the site of injury. Interestingly, the Peptide-treated group had a similar increase in the number of astrocytes at the site of injury compared with the IKVAV-MSP group. However, neurological function was not improved in this group suggesting that IKVAV must be present on the peptide to achieve successful restoration of movement. The reason for similar numbers of astrocytes between these groups is unknown and further investigation into the mechanism of astrocyte induction with the membrane-spanning peptide is warranted. Compelling evidence indicates that formation of a glial scar inhibits axonal regeneration 4 weeks after SCI [8]. In agreement with this finding, we observed functional behavioral scores reached a plateau from week 3 to week 4. Reactive astrocytes divide and slowly migrate into CNS injuries, eventually to fill in the vacant space and most of a glial scar is made up of astrocytes. Whether astrocytes inhibit or promote neuronal growth has been investigated [38]. Multiple models have demonstrated the molecular composition of the glial scar and production of inhibitory molecules by astrocytes are contributing factors for regenerative failure of axons following injury [39–43]. Yet other studies suggest there is a promoting effect of astrocytes on axonal regeneration [8,35,36,44]. The *in vitro* evidence for the ability of astrocytes to promote or inhibit axon regeneration is conflicting. In our study, mice treated with IKVAV and a transmembrane-spanning peptide or a transmembrane-spanning peptide alone, had increases in astrocytes at the site of injury. In contrast to the peptide alone-stimulated astrogliosis without neuronal recovery, IKVAV-MSP treatment produced neuronal recovery which occurred along with astrogliosis. Further research is warranted to determine why this difference in functional outcome with a similar increase in astrocytes occurred. However, treatment success with IKVAV-MSP was definitive by our measures.

A major concern for patients following SCI is skeletal muscle atrophy. In rodent models of SCI, skeletal muscle atrophy is associated with smaller muscle bundles and an increase in intramuscular fat (IMF). Moreover, IMF continues to increase over time in incomplete SCI [45]. IKVAV-MSP treatment prevented muscle atrophy as evinced by increased muscle mass, larger muscle bundle size, and decreased IMF. Further, functional improvement in IKVAV-MSP mice indicates the re-innervation of locomotory muscles.

Our results are significant because none of the mice in our study received physical therapy or exercise of any kind other than being allowed free movement within their cage; yet, mice treated with the IKVAV-MSP did not develop the muscle atrophy and increase in IMF seen in the other treatment groups. Increases in muscle mass are possible even years after injury if the appropriate mode and intensity of exercise are utilized even without spinal cord neuron recovery [46,47]. Unilateral neuromuscular electrical stimulation (NMES) can evoke hypertrophy in the knee extensor and adjacent skeletal muscle groups and is associated with a reduction in IMF in human beings with chronic SCI. However, it seems rebuilding muscles after SCI needs more time in order to obtain the increased muscle mass that occurs with physical therapy.

Time is an important factor for recovery following injury. We followed the mice for 28 days post-SCI using a similar model as Tysseling *et al.* which reported IKVAV PA-injected group displayed significant behavioral improvement at 5 weeks and thereafter, compare to the control group [14]. Surprisingly, treatment with our IKVAV-MSP resulted in significant functional recovery observed approximately 1 week after SCI and reached a plateau in improvement of function at week 4. In addition to marked improvement in mBBB scores at week 1, several mice displayed self mutilation. A total of 13 mice chewed on their feet or tails with the majority of the mice in the IKVAV-MSP and Mannitol treatment groups (6 mice in total; Mannitol: 4 mice; Peptide: 2 mice; and IKVAV: 1 mouse). Self-mutilation indicated to us the mice may have been regaining feeling in their extremities. In addition to this, some of the mice were not able to urinate possibly because SCI is known to interrupt neuronal circuits which control the bladder function [48]. Interestingly, in the last week of the study most of the IKVAV-MSP-treated mice regained their ability to urinate (4/6) suggesting reinnervation with restoration of function [14].

Our observations are similar to kinematic studies of locomotor recovery following SCI in rats with the thoracic clip compression model reporting that during the 6-week postinjury period, a significant amount of spontaneous locomotor recovery occurred in 80% of the rats with a return of well-defined locomotor hind limb pattern. However, substantial residual abnormalities persisted up to 6 weeks after SCI including postural deficits [49].

## Future perspective

Further research using greater numbers of animals and following the recovery and neuron regeneration over a longer period of time such as 3–6 months is warranted to determine whether the improvements seen in the short term using IKVAV-MSP are maintained later in the regenerative process. Additional studies will also likely include electromyography and anterograde tracing to provide additional evidence of neural regeneration. The results suggest a role for cell-spanning peptides as a platform nanotechnology for the treatment of injury or disease.

The use of molecular anchors to present bioactive molecules in tissue and direct regenerative activities of stem cells *in vivo* has the potential to accelerate the development of stem cell therapies. Our findings and our previous work suggest that membrane-spanning peptides could be used to direct signals for regeneration and repair to injured tissues by direct injection into the site or intravenous administration as was previously demonstrated [50]. As the field progresses we anticipate that membrane-spanning peptides could be used to display an array of bioactive molecules capable of directing complex cellular processes.

**Executive summary**

Cell membrane-spanning peptides displaying the isoleucine-lysine-valine-alanine-valine biotope enhance regeneration of injured spinal cord.
This technology has broad application to stem cell-directed therapies that can be used to treat injury and disease.
